# l-cysteine suppresses ghrelin and reduces appetite in rodents and humans

**DOI:** 10.1038/ijo.2014.172

**Published:** 2014-10-14

**Authors:** A K McGavigan, H C O'Hara, A Amin, J Kinsey-Jones, E Spreckley, A Alamshah, A Agahi, K Banks, R France, G Hyberg, C Wong, G A Bewick, J V Gardiner, A Lehmann, N M Martin, M A Ghatei, S R Bloom, K G Murphy

**Affiliations:** 1 Department of Investigative Medicine, Imperial College London, London, UK; 2 AstraZeneca R&D, Mölndal, Sweden; 3 Division of Diabetes & Nutritional Sciences, King's College London, London, UK; 4 NextRx, Gothenburg, Sweden

## Abstract

**Background:**

High-protein diets promote weight loss and subsequent weight maintenance, but are difficult to adhere to. The mechanisms by which protein exerts these effects remain unclear. However, the amino acids produced by protein digestion may have a role in driving protein-induced satiety.

**Methods:**

We tested the effects of a range of amino acids on food intake in rodents and identified l-cysteine as the most anorexigenic. Using rodents we further studied the effect of l-cysteine on food intake, behaviour and energy expenditure. We proceeded to investigate its effect on neuronal activation in the hypothalamus and brainstem before investigating its effect on gastric emptying and gut hormone release. The effect of l-cysteine on appetite scores and gut hormone release was then investigated in humans.

**Results:**

l-Cysteine dose-dependently decreased food intake in both rats and mice following oral gavage and intraperitoneal administration. This effect did not appear to be secondary to behavioural or aversive side effects. l-Cysteine increased neuronal activation in the area postrema and delayed gastric emptying. It suppressed plasma acyl ghrelin levels and did not reduce food intake in transgenic ghrelin-overexpressing mice. Repeated l-cysteine administration decreased food intake in rats and obese mice. l-Cysteine reduced hunger and plasma acyl ghrelin levels in humans.

**Conclusions:**

Further work is required to determine the chronic effect of l-cysteine in rodents and humans on appetite and body weight, and whether l-cysteine contributes towards protein-induced satiety.

## Introduction

High protein diets can drive weight loss and support subsequent weight maintenance.^1,2^ Identifying the mechanisms by which protein drives satiety and weight loss may help identify therapeutic options for the treatment of obesity. Recent work has suggested that the amino-acid products of protein digestion may be sensed peripherally and centrally to regulate appetite.^3^

Amino acids are critical for normal physiological function and many species are able to adapt their protein intake to ensure an adequate supply of essential amino acids.^4^ Different types of protein exert variable effects on appetite,^5, 6, 7, 8^ which may reflect their varied amino-acid constituents. The discovery of amino-acid-sensing G-protein-coupled receptors, and their expression in regions including the gastrointestinal tract, has led to the suggestion that these receptors may sense amino-acid intake to regulate appetite.^9^ Leucine, a branched-chain essential amino acid, reduces food intake by modulating mammalian target of rapamycin activity in the hypothalamus and/or the nucleus tractus solitarius (NTS).^10,11^ Yet, the effect of leucine alone does not account for the success of high-protein diets,^12^ suggesting additional individual amino acids may also contribute. However, the amino acids with anorectic effects and the mechanisms by which they mediate these effects remain to be fully elucidated.

We therefore investigated the effects of oral and intraperitoneal administration of a range of amino acids on food intake in rodents. These studies identified l-cysteine, a conditionally essential amino acid that acts as a precursor for biologically active molecules such as hydrogen sulphide (H2S), glutathione and taurine, as an anorectic agent. We subsequently further investigated the effects of l-cysteine on appetite in rodents and humans and the mechanisms mediating these effects.

## Materials and methods

### Animals

Male Wistar rats (8–10 weeks, 220–250 g, Charles River, Margate, UK) and male C57BL/6 mice (8–10 weeks, 22–25 g, Harlan, Bicester, UK) were maintained in individual cages under controlled temperature (21–23 °C) and light (12:12 light–dark cycle, lights on at 0700 hours) with *ad libitum* access to food (RM1 diet; SDS, Witham, UK) and water unless otherwise stated. Transgenic ghrelin-overexpressing mice were generated as previously described.^13^ GPRC6A knockout mice were obtained from the Knockout Mouse Project Repository (supplementary material). All animal procedures were approved under the British Home Office Animals (Scientific Procedures) Act 1986 (Project Licence 70/6402 or 70/7236).

Male Wistar rats used in subdiaphragmatic vagal deafferentation (SDA) experiments were maintained in individual cages under controlled temperature (21–23 °C) and light (12:12 light–dark cycle, lights on at 0600 hours) with *ad libitum* access to food (R70, Lactamin, Sweden) and water unless otherwise stated. Experiments were approved by the Gothenburg Animal Review Board (ethical application number 101505).

### Feeding studies

Animals were orally gavaged or intraperitoneally (IP) injected with vehicle or l-amino acids during the early light phase following an overnight 16-h fast. Food intake was measured at 1 h post administration with any notable spillage accounted for.

### Effect of l-cysteine on behaviour and conditioned taste aversion

Behavioural studies were used to investigate the possibility that the administration of l-cysteine and the associated reduction in food intake was secondary to nonspecific behavioural effects. To confirm that l-cysteine did not result in aversive effects, we also investigated whether oral administration of l-cysteine at 1, 2 or 4 mmol kg^−1^ resulted in conditioned taste aversion (CTA) using an established method^14^ (see Supplementary Material).

### Effect of l-cysteine on energy expenditure

The effect of 2 mmol kg^−1^ l-cysteine on activity and energy expenditure was investigated using a 24-chamber open-circuit Comprehensive Laboratory Animal Monitoring System (CLAMS; Columbus Instruments, Columbus, OH, USA)^15^ (see Supplementary Material).

### The role of downstream metabolites and the *N*-methyl d-aspartate (NMDA) receptor in mediating the anorectic effect of l-cysteine

l-cysteine is metabolised via a number of pathways (Supplementary Figure 2A). l-cysteine and some of its metabolites have been reported to act as weak NMDA receptor agonists.^16^ The roles of cysteine metabolites and the NMDA receptor in l-cysteine-induced hypophagia and food intake were therefore investigated (see Supplementary Material).

### Effect of l-cysteine on cFos immunoreactivity

**Figure 1:**
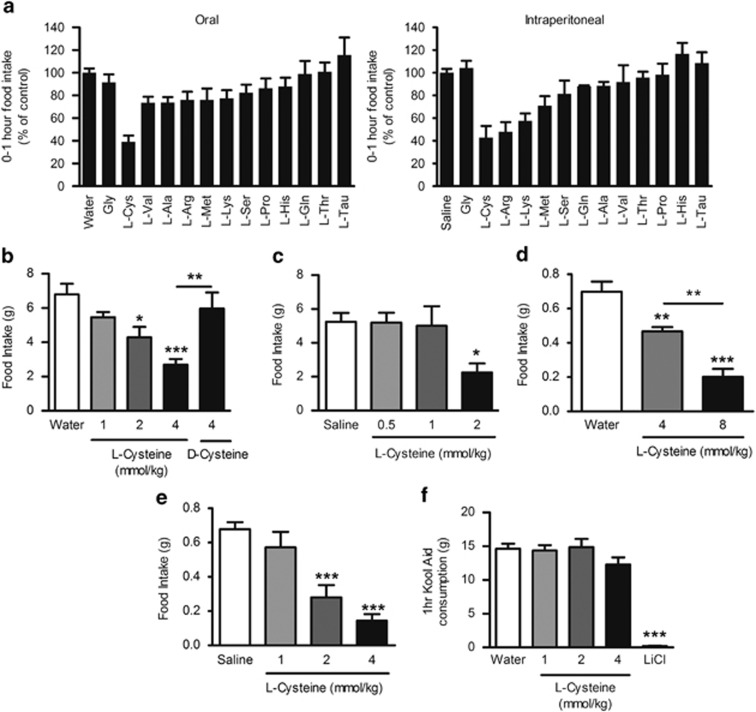
The effect of l-cysteine on food intake in rodents. (**a**) The effect of oral gavage administration of 4 mmol kg^−1^ (left panel) and of intraperitoneal administration of 2 mmol kg^−1^ (right panel) l-amino acids on 0–1-h food intake in the early light phase following an overnight fast, *n*=5–10. (**b**) The effect of oral administration of l- and d-cysteine and (**c**) the effect of intraperitoneal administration of l-cysteine on 0–1-h food intake during the early light phase after an overnight fast in male Wistar rats (*n*=6–8), and (**d**) the effect of oral and (**e**) intraperitoneal administration of l-cysteine on 0–1 h food intake during the early light phase after an overnight fast in male C57BL/6 mice (*n*=7–10). (**f**) The effect of oral administration 1, 2 and 4 mmol kg^−1^ l-cysteine and 127 mg kg^−1^ LiCl on conditioned 1 hour KoolAid consumption in male Wistar rats (*n*= 5–9). All data are expressed as mean±s.e.m. *P<0.05, **P<0.01, ***P<0.001.

Rats were fasted overnight before receiving an oral gavage of water, 4 mmol kg^−1^ l-cysteine or 4 mmol kg^−1^ glycine (*n*=4–6). Glycine was used as a negative control as it was previously found to have no effect on food intake (Figure 1a). Two animals were IP injected with hypertonic saline as positive controls for the staining procedure.

Transcardial perfusion, tissue preparation and immunohistochemistry were carried out as previously described,^17^ with animals killed 90 min post gavage.

Cell bodies positive for cFos-like immunoreactivity (cFLI) were counted bilaterally from matched sections in hypothalamic and brainstem nuclei by an observer blinded to the treatment. Nuclei were defined in relation to anatomical landmarks according to the rat brain atlas of Paxinos and Watson.^18^

### Gastric emptying

Gastric emptying was measured using an established method.^19,20^ Rats were fasted overnight, then received an intraperitoneal injection of saline, 2 mmol kg^−1^ l-cysteine, 2 mmol kg^−1^ glycine (negative control) or 10 nmol kg^−1^ A71623 (cholecystokinin (CCK)-A receptor agonist, positive control) (*n*=4–7) followed immediately by an oral gavage of 2 ml of a 1.5% methylcellulose (4000cP), 0.05% phenol red solution. Animals were culled by decapitation 30 min later and the stomach removed for quantification of the remaining phenol red.

Using the same method, the effect of l-cysteine on gastric emptying was further investigated in mice. Mice were fasted overnight, then received an IP injection of saline, 0.5 mg kg^−1^ devazepide, 10 nmol kg^−1^ A71623, 0.5 mg kg^−1^ devazepide and 10 nmol kg^−1^ A71623, 2 mmol/kg l-cysteine, or 0.5 mg kg^−1^ devazepide and 2 mmol kg^−1^ l-cysteine followed by 0.2 ml of the 1.5% methylcellulose, 0.05% phenol red solution.

### Subdiaphragmatic vagal deafferentation surgery

Rats were adapted to a nutritionally complete liquid diet (Nestlé Nutrition, Resource Energy, 1.5 kcal ml^−1^) for 3 days before undergoing SDA or sham surgery. SDA surgery involved left intracranial rhizotomy and transection of the dorsal subdiaphragmatic trunk of the vagus, resulting in 50% deafferentation and complete subdiaphragmatic vagal deafferentation.^21,22^ Post surgery, rats received liquid diet for 2 days and then a semiliquid diet for 4 days, and were given 10 days to fully recover. The effect of oral gavage of 4 mmol kg^−1^ l-cysteine on food intake was then measured.

### Effect of l-cysteine on gut hormone release

Rats were fasted overnight before receiving an oral gavage of water or 4mmol kg^−1^ l-cysteine (*n*=7–8) or intraperitoneal injection of saline or 2 mmol kg^−1^ l-cysteine (*n*=6–8). Animals were returned to their home cages and 30 min post administration were culled by decapitation and trunk blood collected in lithium heparin tubes containing 0.6 mg aprotinin. Plasma was separated by centrifugation and then frozen and stored at −20 °C for analysis. After centrifugation an aliquot of plasma was acidified with HCl to a concentration of 1N before freezing.

Plasma glucagon-like peptide-1 (GLP-1) and peptide YY (PYY) were measured using established in-house radioimmunoassays,^23,24^ and acyl ghrelin using a commercially available enzyme immunoassay (SPI Biobertin, SPI bio bertin, Bertin Pharma, Montigny le Bretonneux, France).

### Repeated administration

Adult male Wistar rats were orally gavaged three times throughout the dark phase (at 1900, 2300 and 0300 hours) with water, 4 mmol kg^−1^ l-cysteine or 4 mmol kg^−1^ glycine (negative control) (*n*=6–9). Body weight and food intake were measured daily at the onset of the dark phase.

Male C57BL/6 mice aged 6 weeks were maintained in group housing with *ad libitum* access to high-fat diet (D12492, Research Diets, New Brunswick, USA; containing 60% of its energy as fat) and water for 14 weeks, reaching an average weight of 39.1 g. Animals were then individually housed and allowed 1 week to acclimatise before experiments commenced as for rats above (*n*=8–10).

### Clinical studies

#### Study participants

Human studies were conducted following ethical approval (West London Research Ethics Committee 1, London, UK) and according to the principles of the Declaration of Helsinki. All participants gave their written informed consent before study enrolment.

Healthy male (*n*=2, 1 Caucasian, 1 South Asian) and female (*n* = 5, all Caucasian) subjects with a mean (±s.d.) age of 35.9±10.9 years and body mass index of 23.7 ± 4.3 kg m^−2^ who had been weight stable for the 3 months were recruited.

#### Study design

Participants attended three study visits and reported to the clinical research facility at 0830 hours having fasted from 2100 hours  the night before. On each visit, participants were cannulated in the antecubital fossa for serial blood sampling and asked to consume a 200- ml drink containing either vehicle alone, 200 ml containing 0.07 g kg^−1^ l-cysteine or 200 ml containing 0.07 g kg^−1^ glycine in a single-blind (participant) randomised order. Blood samples were collected at 15- min intervals starting at *t*=−15 min for 2.5 h after dosing. Participants were asked to complete visual analogue scales (VAS) at each time point to assess hunger, fullness, nausea, anxiety, irritability and sleepiness. Participants were asked to report any additional side effects during and after the visits. Baseline plasma and serum was assayed for routine clinical chemistry (liver and kidney function, calcium and electrolytes). Plasma acyl ghrelin was measured using a commercially available ELISA (Merck Millipore, MA, USA).

### Statistical analysis

Acute food intake and area under the curve (AUC) data is expressed as mean±s.e.m. and was analysed by one-way analysis of variance (ANOVA) and *post hoc* Tukey’s test. CTA data were analysed using one-way ANOVA with *post hoc* Dunnett's test. Data from transgenic mice were analysed by paired *t*-test. CLAMS data, cumulative data and data from GPRC6A knockout mice were analysed by two-way repeated measures ANOVA with a *post hoc* test with Bonferroni correction. Behavioural data were analysed by Mann–Whitney test and cFos data were analysed by Kruskal Wallis with Dunn’s *post hoc* comparison.

All additional methods are included in Supplementary Material.

## Results

### The effect of l-amino acids on food intake in rats

To investigate the anorectic potential of specific l-amino acids *in vivo*, the effect on food intake following oral and intraperitoneal administration of a range of amino acids was examined in rats. Of the amino acids investigated, l-cysteine reduced food intake to the greatest extent following both oral and intraperitoneal administration (Figure 1a). We therefore decided to further investigate the effects of l-cysteine on food intake.

### l-cysteine decreases food intake in rodents

Oral administration of l-cysteine dose-dependently decreased food intake in rats and mice (Figure 1b and d). Food intake following 4 mmol kg^−1^ l-cysteine was significantly reduced, compared with food intake following saline or 4 mmol kg^−1^ d-cysteine 0–1 h following administration (*P*<0.01) in rats, demonstrating an enantiomer-specific effect (Figure 1b). Intraperitoneal administration of l-cysteine also dose-dependently decreased food intake in rats and mice (Figures 1c and e).

### l-Cysteine increases respiratory exchange ratio (RER) in mice

A single IP injection of 2 mmol kg^−1^ l-cysteine in the early light phase during food restriction significantly increased RER from the first 30–90 min post administration (*P*<0.001), and significantly reduced ambulatory activity during the first 30 min post injection (XTOT: *P*<0.001, XAMB: *P*<0.01, ZTOT: *P*<0.05) (Supplementary Figure 1).

### l-cysteine does not induce aversive behaviour in rodents

Oral administration of 4 mmol kg^−1^ l-cysteine to rats significantly reduced feeding behaviour (*P*<0.05) (Supplementary Table 1) without altering behaviours indicative of illness or nausea. Intraperitoneal administration of 2 mmol kg^−1^ l-cysteine to rats and mice did not cause any behaviour indicative of illness or nausea compared with control (Supplementary Tables 2 and 3). Oral gavage administration of l-cysteine at doses up to 4 mmol kg^−1^ did not cause CTA in rats (Figure 1f). In addition, our data suggested that l-cysteine does not mediate its anorectic effects via the NMDA receptor, GPRC6a or via downstream metabolites (see supplementary results and Supplementary Figures 2 and 3).

### l-cysteine increases neuronal activation in the rat brainstem

**Figure 2:**
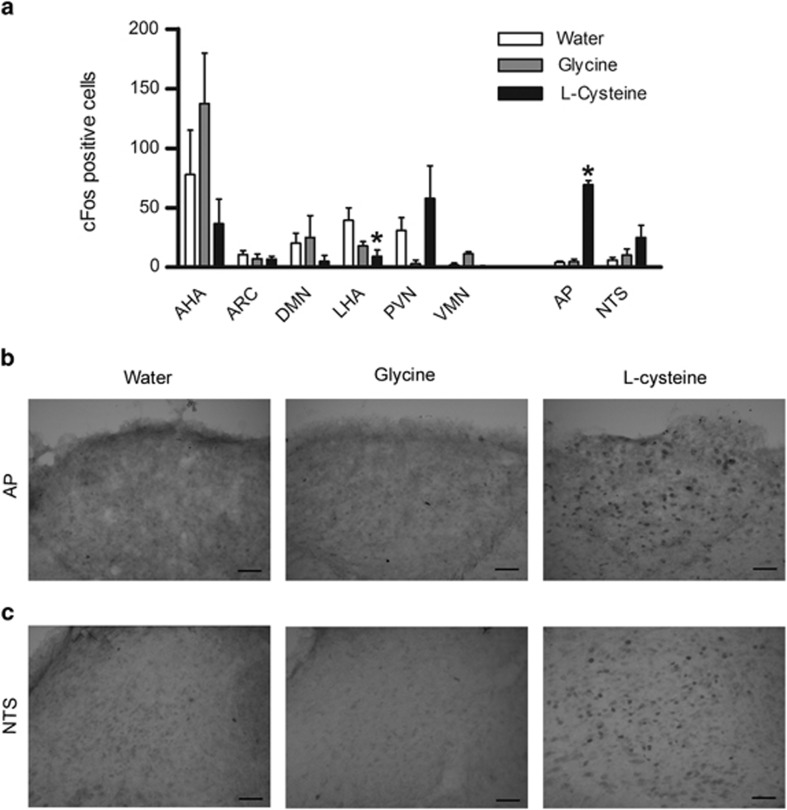
The effect of oral administration of l-cysteine on hypothalamic and brainstem cFos expression in rats. (**a**) Number of cFos-positive cells in the hypothalamic nuclei: AHA, ARC, DMN, LHA, PVN, VMN and brainstem nuclei: AP and NTS following oral gavage of water, 4 mmol kg^−1^ glycine or 4mmol kg^−1^ l-cysteine (*n*=3–6), data expressed as median and interquartile range, *P<0.05. (**b**) Representative sections of the AP (l-r: water, glycine, l-cysteine at −14.0 mm posterior from bregma) and (**c**) NTS (l-r: water, glycine, l-cysteine at −13.3.mm posterior from bregma), scale bar=50 μm.

Oral administration of 4 mmol kg^−1^ l-cysteine significantly reduced cFLI in the lateral hypothalamic area (LHA) (*P*<0.05) (Figure 2a). However, there was no significant difference in cFLI in the LHA between animals treated with l-cysteine and glycine (used as a negative control) (Figure 1a), suggesting the reduction in cFLI in the LHA was not specifically related to the anorectic effects of l-cysteine. Oral administration of 4mmol kg^−1^ l-cysteine significantly increased cFLI in the area postrema compared with water-treated controls (*P*<0.05) (Figure 2a, representative sections Figure 2b). There was a trend for increased cFLI in the NTS (Figure 2a, representative sections Figure 2c).

To investigate whether l-cysteine mediated its effect on food intake centrally, we measured food intake following administration of l-cysteine into the lateral ventricle. Central anorectic doses resulted in severe behavioural abnormalities, including seizure-like activity. Such behavioural effects were not previously observed following oral or IP administration of anorectic doses (Supplementary Tables 1–3). We therefore hypothesised that the reduction in food intake following central administration of high doses of l-cysteine was secondary to these behavioural abnormalities and that these effects were caused by high central concentrations of l-cysteine activating central NMDA receptors. Accordingly, NMDA receptor antagonism completely blocked the reduction in food intake and the behavioural abnormalities observed following l-cysteine administration (*P*<0.05) (Supplementary Figure 4B).

### l-cysteine reduces gastric emptying

**Figure 3:**
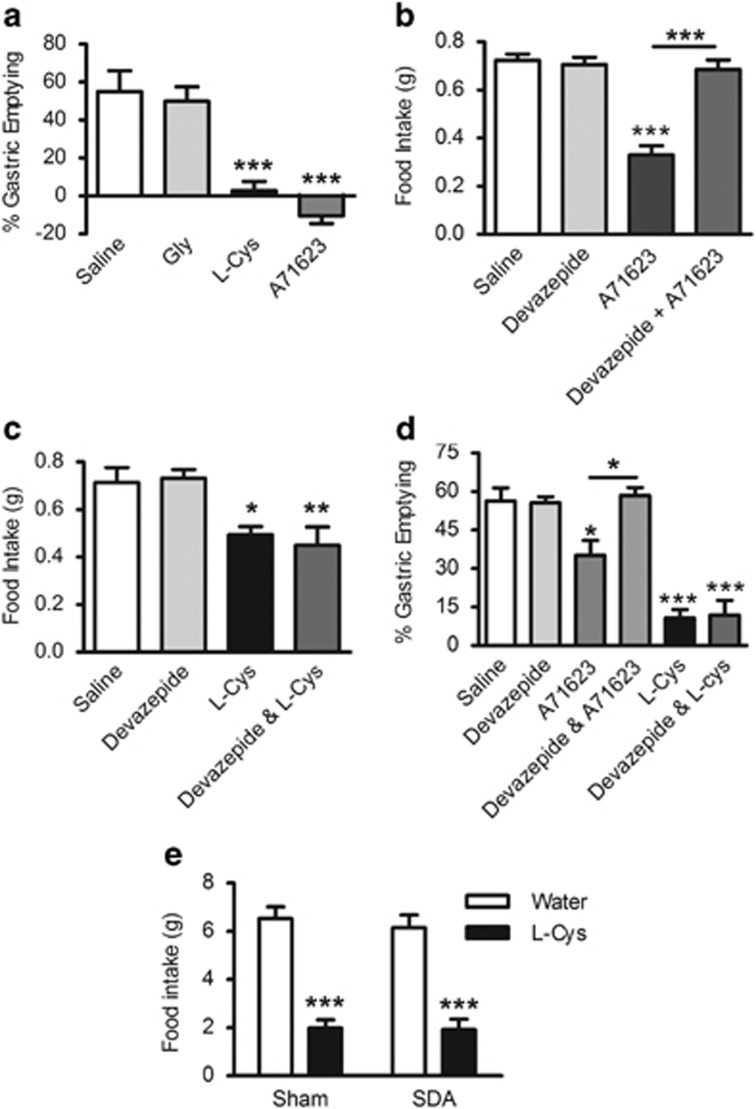
The effect l-cysteine on gastric emptying and the role of the CCK-1R and vagal afferents. (**a**) The effect of IP administration of saline, 2 mmol kg^−1^ l-cysteine, glycine or 10 nmol kg^−1^ A71623, a CCK-1 receptor agonist, on gastric emptying of 2 ml of a semi-solid non-nutritive substance given by oral gavage in rats 30 min post injection relative to gastric emptying at time 0 (*n*=4–7). (**b**) The effect of IP administration of 0.5 mg kg^−1^ devazepide on the anorectic effect of 10 nmol kg^−1^ A71623 in the 0–1 h period post administration (*n*=8–9). (**c**) The effect of 0.5 mg kg^−1^ devazepide on the anorectic effect of 2 mmol kg^−1^ l-cysteine in the 0–1 h period post administration (*n*=8–9). (**d**) The effect of devazepide on the l-cysteine-induced delay in gastric emptying in mice (*n*=4–7). (**e**) The effect of oral gavage of water and 4 mmol kg^−1^ l-cysteine on 0–1 h food intake (*n*=9–10) in male Wistar rats having undergone sham or subdiaphragmatic vagal deafferentation (SDA). Data expressed as mean±s.e.m. *P<0.05, **P<0.01, ***P<0.001.

Gastric emptying and gastric distension can affect satiety. Therefore, we investigated the effect of l-cysteine on gastric emptying. Intraperitoneal administration of 2 mmol kg^−1^ l-cysteine significantly reduced gastric emptying 30 min after administration to rats (*P*<0.001) (Figure 3a). Administration of 2 mmol kg^−1^ glycine, used as a negative control, did not affect gastric emptying. To investigate whether CCK, a potent inhibitor of gastric emptying, was responsible for the reduction in food intake and delayed gastric emptying, following administration of l-cysteine, a CCK-1 receptor antagonist was used. The CCK-1 receptor antagonist devazepide (0.5 mg kg^−1^) inhibited the effect of the CCK-1 receptor agonist, A71623, on food intake and gastric emptying in mice (Figures 3b and d) but did not inhibit the effect of l-cysteine on food intake or gastric emptying (Figures 3c and d). The degree of gastric distension is primarily communicated to the brain through vagal afferents. The role of vagal afferents in mediating the effect of l-cysteine on food intake was investigated in rats that had undergone subdiaphragmatic vagal deafferentation. There was no significant difference in the anorectic response of sham and SDA animals following oral administration of l-cysteine, suggesting vagal afferents are not essential for the anorectic effects of l-cysteine (Figure 3e).

### l-cysteine suppresses plasma ghrelin

**Figure 4:**
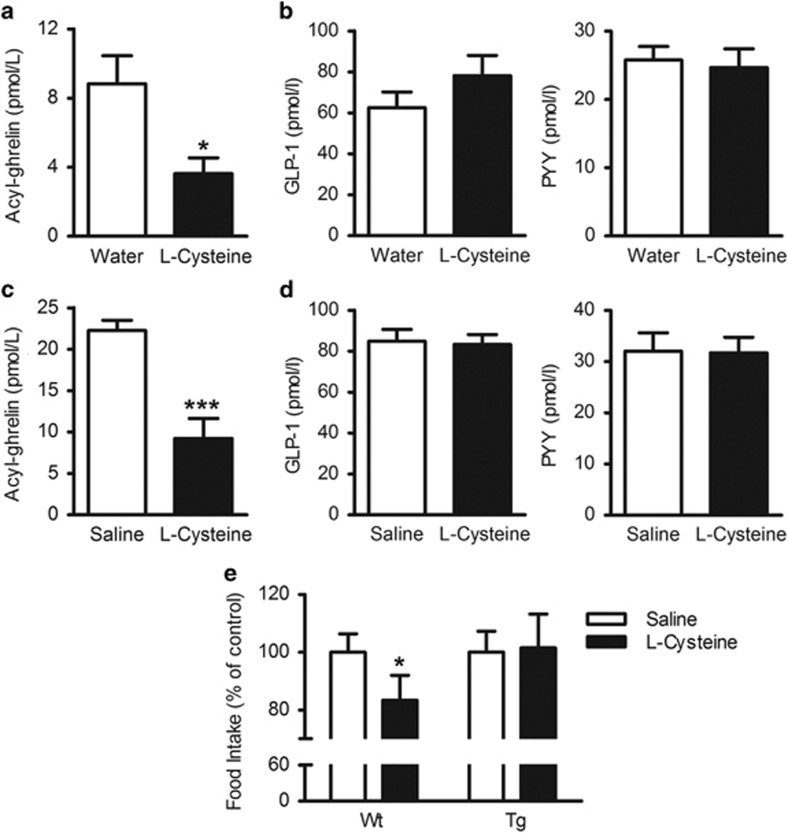
l-cysteine suppresses plasma acyl ghrelin levels in rats. Plasma levels of (**a**) acyl ghrelin and (**b**) l-r: GLP-1 and PYY, 30 min after oral gavage of water or 4 mmol kg^−1^ l-cysteine (*n*=7–8), (**c**) acyl ghrelin and (**d**) l-r: GLP-1 and PYY, 30 min after intraperitoneal administration of saline or 2 mmol kg^−1^ l-cysteine (*n*=6–8). (**e**) The effect of 2 mmol kg^−1^ l-cysteine on 0–1-h food intake in wild-type and transgenic ghrelin-overexpressing mice after an overnight fast (*n*=15–21). Data expressed as mean±s.e.m. *P<0.05, **P<0.01, ***P<0.001.

Thirty minutes after oral administration of 4 mmol kg^−1^ l-cysteine, plasma levels of acyl ghrelin were significantly reduced compared with water-treated rats (*P*<0.05) (Figure 4a). IP administration of 2 mmol kg^−1^ l-cysteine also significantly reduced plasma acyl ghrelin levels compared with saline-treated animals (*P*<0.001) (Figure 4c), but not GLP-1 or PYY levels following oral (Figure 4b) or IP (Figure 4d) administration. l-cysteine did not suppress food intake in transgenic ghrelin-overexpressing mice (Figure 4e).

### l-cysteine suppresses hunger and plasma ghrelin in humans

**Figure 5:**
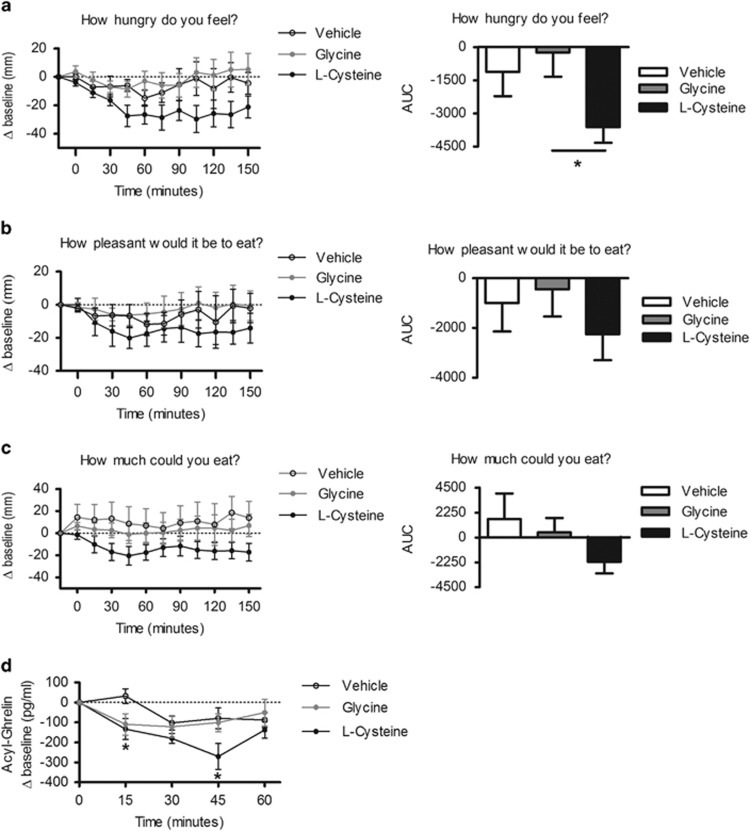
l-cysteine suppresses appetite and ghrelin release in humans. (**a**–**c**) Visual analogue scales and area under the curve following ingestion of vehicle, 0.07 g kg^−1^ l-cysteine or 0.07 g kg^−1^ glycine (*n*=7) (**d**) The change in plasma ghrelin following oral ingestion of vehicle, 0.07 g kg^−1^ l-cysteine or 0.07 g kg^−1^ glycine (*n*=7). Data are expressed as mean±s.e.m. *P<0.05.

l-cysteine reduced feelings of hunger compared with glycine-treated controls as measured by visual analogue scales (VAS) (*P*<0.05) (Figure 5a). There was a trend for a decrease in VAS scores for ‘How pleasant would it be to eat’ (Figure 5b) and ‘How much could you eat’ (Figure 5c). l-cysteine significantly reduced plasma acyl ghrelin levels at 45 min post administration compared with levels following vehicle and glycine treatment (*P*<0.05) (Figure 5d), the time point at which the largest cysteine-induced change from baseline for ‘How pleasant would it be to eat’ and ‘How much could you eat’ occurred. l-cysteine had no effect on plasma GLP-1 and PYY (Supplementary Figure 5). No significant side effects were reported during or after the study (Supplementary Figure 6).

### Repeated administration of l-cysteine reduces cumulative food intake

**Figure 6:**
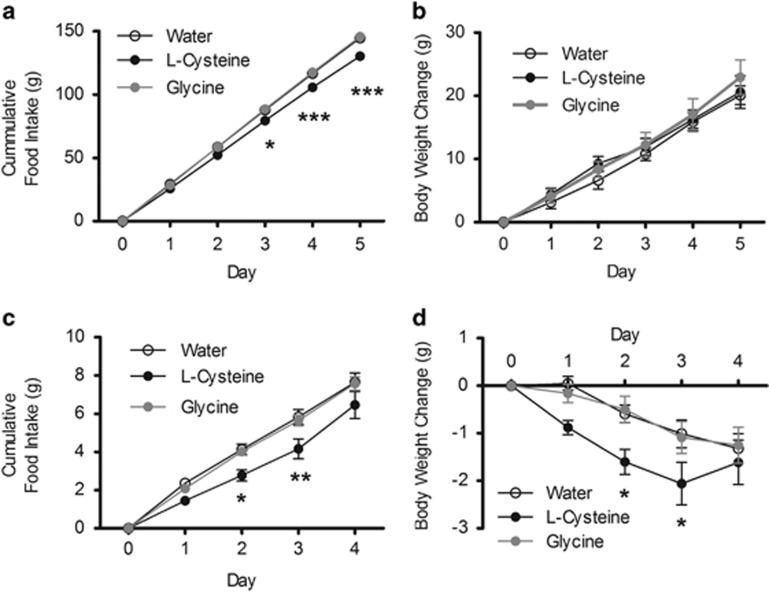
The effect of repeated administration of l-cysteine in lean rats and diet-induced obese (DIO) mice. (**a**) The effect of 3 × daily oral gavage of water, 4 mmol kg^−1^ l-cysteine or 4 mmol kg^−1^ glycine on cumulative food intake and (**b**) body weight change in lean Wistar rats (*n*=6–9), (**c**) on cumulative food intake and (**d**) body weight change in DIO C57BL/6 mice (*n*=8–10). Data are expressed as mean±s.e.m. *P<0.05, **P<0.01, ***P<0.001.

Our data demonstrated that l-cysteine could acutely suppress appetite. We subsequently investigated whether the anorectic effect of l-cysteine was sustained following repeated administration in rodents. Repeated administration of l-cysteine over a period of five nights significantly reduced cumulative food intake in lean rats compared with water and glycine-treated controls (*P*<0.001) (Figure 6a). However, this change in food intake did not result in a significant difference in body weight gain between the groups (Figure 6b).

To investigate whether l-cysteine could reduce body weight in an obese model, we used the same protocol in diet-induced obese (DIO) mice. l-cysteine-treated animals had lost significantly more weight than water and glycine-treated controls on days 2 and 3 (*P*<0.05) (Figure 6d). Cumulative food intake was also significantly lower in the l-cysteine group on days 2 (*P*<0.05) and 3 (*P*<0.01) (Figure 6c).

## Discussion

Our data identify a novel anorectic effect for the amino acid l-cysteine. l-cysteine reduced food intake in rodents and hunger in a small scale study in humans, and reduced plasma levels of the orexigenic gut hormone acyl ghrelin in both rodents and humans.

Jordi *et al.*^25^ recently described the effects of an oral dose of 6.7 mmol kg^−1^ of the 20 proteinogenic amino acids on food intake, and identified l-arginine, l-lysine and l-glutamic acid as the most anorectic amino acids. However, our data suggest that at the lower doses we used (oral gavage: 4 mmol kg^−1^, intraperitoneal: 2 mmol kg^−1^), l-cysteine is more anorectic than l-arginine and l-lysine. We found that l-cysteine reduced food intake in a dose-dependant manner. The amounts of l-cysteine administered were higher than a rodent would be expected to consume in a single bout of eating, and thus represent a pharmacological effect. If l-cysteine does have a physiological effect on appetite, then it is likely to act in concert with other products of protein digestion, and thus the effects of l-cysteine *per se* may be difficult to detect. However, if the precise mechanisms mediating the anorectic effects of l-cysteine are characterised, it would be interesting to investigate whether blocking these mechanisms can inhibit protein-induced satiety and the long-term metabolic effects of a high protein diet.

l-cysteine was effective at doses that did not induce conditioned taste aversion or evoke abnormal behaviour. These data suggest that l-cysteine does not result in unpleasant post-ingestive consequences that might result in a nonspecific reduction in food intake. However, there may be an effect of l-cysteine at the highest dose tested in the CTA protocol, though it does not achieve statistical significance, and the post-injection increase in locomotor activity observed in saline-treated mice in the CLAMS cages was suppressed in cysteine-treated mice, which may suggest a degree of treatment associated discomfort. Further work would thus be useful to determine whether higher doses of l-cysteine are associated with aversive effects. An isomolar dose of d-cysteine did not reduce food intake following oral or intraperitoneal administration. This l-enantiomer specificity may indicate a potential role for promiscuous amino-acid-sensing receptors such as T1R1/T1R3, CaSR and GPRC6A, which are reported to be activated by l- but not d-amino acids.^26, 27, 28^ However, l-cysteine reduced food intake in GPRC6A knockout mice to the same extent as wild type, suggesting GPRC6A is not necessary for the anorectic effects of l-cysteine. l-cysteine induces a strong T1R1/T1R3-mediated cellular response *in vitro*, but other amino acids that also induce a strong response, such as serine and threonine,^26^ did not have significant effects on food intake. l-cysteine can also activate the CaSR. However, histidine is reported to induce the strongest CaSR-mediated cellular response of the proteinogenic amino acids, but did not reduce food intake in our studies. These data suggest that the promiscuous amino-acid receptors T1R1/T1R3, CaSR or GPRC6A are unlikely to mediate the effects of l-cysteine on appetite, though further studies are needed to conclusively demonstrate that T1R1/T1R3 and CaSR are not involved.

l-cysteine increased the number of cFos-positive cells in the AP, suggesting the brainstem may mediate its effects on food intake. l-cysteine reduced gastric emptying via a CCK-1-receptor-independent mechanism and reduced food intake independently of the CCK-1 receptor and vagal afferents. These results accord with those published by Jordi *et al.*^25^ for the amino acids l-arginine and l-glutamic acid, suggesting that there may be similarities between the mechanisms by which these amino acids and l-cysteine mediate their anorectic effect.

l-cysteine may also work by modulating gastrointestinal hormone release. l-cysteine did not alter circulating PYY or GLP-1 concentrations in rodents or humans. The assays used measured total PYY and GLP-1 immunoreactivity, and it is thus possible that specifically measuring the active forms of these hormones might have revealed an effect, though the levels of these active forms generally correlate with the total circulating concentrations.^29,30^ l-Cysteine did reduce circulating plasma acyl ghrelin levels in both rodents and humans. This reduction in ghrelin coincided with the greatest decrease in appetite in humans, and the effect of l-cysteine on food intake was attenuated in transgenic ghrelin overexpressing mice. The mechanism regulating ghrelin secretion remains unclear. However, other hormones, including bombesin, somatostatin, CCK, GLP-1 and insulin, have all been linked to the suppression of acyl ghrelin release. The data presented in this paper suggest that the effect of l-cysteine on food intake does not involve GLP-1 or CCK. Interestingly, the CaSR has recently been localised to X/A cells, where it can have both inhibitory and stimulatory actions on ghrelin release.^31^

Our data demonstrate that a single dose of l-cysteine can reduce appetite in rodents, and can reduce subjective feelings of appetite in a small scale human study. These effects may, at least in part, be mediated by a reduction in circulating plasma levels of the orexigenic hormone acyl ghrelin. To assess whether this reduction in appetite could be maintained and potentially modulate body weight we investigated the effect of repeated administration in lean rats and obese mice. A previous study has demonstrated that supplementing the diet of rats with 1 or 2% l-cysteine can reduce food intake and body weight, though this study did not investigate whether these effects might be mediated by an aversion to the taste of the supplemented diets.^32^ Repeated administration of l-cysteine significantly reduced cumulative food intake in lean animals but had no significant effect on body weight over the duration of the study. To determine whether l-cysteine could modulate body weight under obesogenic conditions we investigated the effect of repeated administration of l-cysteine in DIO mice. l-cysteine caused an initial modest and statistically significant decrease in food intake and body weight in DIO mice. However, the magnitude of this effect appeared to decrease after 3 days of treatment. Weight loss was also observed in control groups, suggesting the administration protocol may have induced undue stress. It is possible that alternative administration protocols might result in more sustained and reliable effects on food intake and body weight. However, higher doses of cysteine were associated with toxicity. As previously mentioned, l-cysteine can have NMDA-receptor-mediated excitatory actions. Dose toxicity studies of l-cysteine have previously been published demonstrating toxic effects after 28 day intravenous administration of 1000 mg kg^−1 ^day^−1^.^33^ Our acute studies used doses that were significantly less than this and our data suggest they were well tolerated.

It has been reported that circulating levels of l-cysteine and its oxidised forms correlate with body mass index and obesity.^34^ However, it is unclear whether this is a causal or consequential factor, and whether the observed differences in circulating l-cysteine reflect differences in cysteine intake or metabolism. A previous study has shown that supplementing rats with l-cysteine can prevent the weight loss associated with a methionine-deficient diet, which may reflect an attenuation of the changes to protein metabolism that this diet results in. This study did not, however, find that supplementation with cysteine administration increased the body weight of rats on a nutrient complete diet.^35^ Cysteine supplementation has also been reported to lessen the age-related decline in food intake in rats, suggesting an appetite stimulating effect in older animals.^36^ Our animals, though adult, were still growing and it is possible that this may also influence their response to l-cysteine. Collectively, these studies suggest that cysteine may have different effects on food intake dependent on the nutritional status and age of the animals. Cysteine administration and dietary supplementation have also been reported to have beneficial effects on glucose levels and insulin sensitivity,^37,38^ though, conversely, l-cysteine has also been shown to have inhibitory effects on glucose-stimulated insulin release from pancreatic β-cells *in vitro*.^39^ Our studies found that l-cysteine transiently increased RER, suggesting cysteine stimulates glucose utilisation and reduces fat utilisation. It would be interesting in future studies to further investigate the effects of l-cysteine on glucose homoeostasis in animals and man.

In summary, our studies identify l-cysteine as an amino acid with potent acute anorectic effects. Further work is required to investigate whether the mechanisms responsible for these effects can be exploited therapeutically.
