# Supplementary material for: l-cysteine suppresses ghrelin and reduces appetite in rodents and humans
Source: Int J Obes (Lond). 2014 Oct 14;39(3):447–55. doi: 10.1038/ijo.2014.172 (PMC4276721; doi:10.1038/ijo.2014.172)
Supplement: Supplementary Information [file ijo2014172x1.doc]

**Supplementary Material**

**Supplementary Methods**

**GPRC6A mice.** GPRC6A-targeted heterozygous C57B1/6N mice were obtained from the Knockout Mouse Project (KOMP) Repository.   Specifically, in this strategy GPRC6A is completely replaced by a ZEN-Ub1 promoter-driven expression selection cassette within a BacVec targeting vector (Velocigene ID: 326L19). ZEN-Ub1 contains a lacZ-galactosidase coding sequence and a neomycin-resistance gene downstream of a human UBIQUITIN C gene [lacZ-(pA)-hUbiPro-neo-p(A)]. The hUbiPro-neo-p(A) sequence is flanked by LoxP sites but there is no conditional targeting potential. The targeting vector was designed to cause both a deletion of the GPRC6A target gene sequence and the insertion of a neomycin-resistance selectable marker, such that the reading frame of the protein coding sequence is interrupted. The deletion size consisted of 16,596 base pairs, which completely covers the GPRC6A locus. Heterozygous mice were bred to produce homozygous GPRC6A-KO animals.

**Behavioural studies**. Rats were fasted overnight and then received an oral gavage of water or 4mmol/kg L-cysteine in the early light phase. Following administration, rats were returned to their home cages, and their food returned. Behaviour was monitored as previously described . Briefly for 1 hour post-administration each animal was observed for 15 sec every 5 min. Each 15 sec observation period was subdivided into 3x5sec observations such that each animal had a total of 36 observations in 1 hour. Behaviour was classified into one of 6 categories: feeding (including drinking), locomotion (including rearing, climbing, burrowing and bed making), grooming, resting (stationary position or sleeping), pica or head-down (abnormal body posture or involuntary movements). This protocol was repeated in rats following IP administration of saline or 2mmol/kg L-cysteine and also in mice following IP administration of saline or 2mmolkg L-cysteine.

**Conditioned taste aversion.** CTA was investigated in response to oral gavage of water, 1, 2 or 4mmol/kg L-Cysteine (n=8-9) or 127mg/kg Lithium Chloride (n=5) as a positive control. Animals were trained daily for 1 week to consume their daily fluid intake within a 1 hour period; at all other times water access was restricted. Any animal failing to consume at least 40ml/kg/24hr on two consecutive days within the training period or showing signs of dehydration was removed from the study. On day 1, 3 and 5 of the subsequent test week, animals were introduced to a novel flavour; Grape Kool-Aid (Northfield, IL, USA) diluted according to manufacturer’s instructions, during the 1 hour fluid access period in place of water followed immediately by the test substance on day 1 and 3. On day 5 Kool-Aid fluid intake was measured but animals were not subject to treatment with the test substances.

**Effect of L-cysteine on energy expenditure.** The effect of 2mmol/kg L-cysteine on activity and energy expenditure was investigated using a 24 chamber- open circuit Comprehensive Laboratory Animal Monitoring System (CLAMS; Columbus Instruments, Columbus, OH). Mice were acclimatized to their cages for 24 hours then food-deprived overnight before being IP injected with either saline or 2mmol/kg L-cysteine. Animals remained fasted for 8 hours post-injection for energy expenditure measurements. Metabolic parameters: oxygen consumption (VO_2_) and carbon dioxide production (VCO_2_) were measured by indirect calorimetry with exhaust air from each cage being sampled sequentially for 1 minute. One sample was taken from each cage approximately every 28minutes. Respiratory Exchange Ratio (RER) was calculated by dividing VCO_2_ by VO_2_. Ambulatory activity on the X axis (XAMB), total beam breaks on the X axis (XTOT) and total beam breaks on the Z axis (ZTOT) were measured simultaneously for each animal using the optical beam technique (Opto M3, Columbus Instruments) .

**The role of downstream metabolites and the NMDA receptor in mediating the anorectic effect of L-cysteine.** L-cysteine and some of its metabolites have been reported to act as weak NMDA receptor agonists . To investigate the role of the NMDA receptor in L-cysteine-induced hypophagia, food intake was measured following pre-treatment with a dose of the NMDA receptor antagonist MK-801 previously shown to block NMDA receptor specific effects, including NMDA-mediated anorectic effects . Mice were fasted overnight then IP injected in the early light phase with saline or 10µg/kg MK-801, then 30 minutes later IP injected with saline or 2mmol/kg L-cysteine and their food returned.

L-cysteine is metabolised via a number of pathways (summarised in Supplemental Fig 2A). To investigate whether the anorectic effect of L-cysteine was mediated via one of the metabolites or was L-cysteine specific, studies inhibiting the enzymes involved in its breakdown were performed (see supplementary material).

In study 1, mice were fasted overnight then IP injected with saline, 1mmol/kg buthionine sulfoximine (BSO), a γ-glutamyl cysteine synthetase (γGCS) inhibitor, 2mmol/kg L-cysteine or BSO and L-cysteine. In study 2, mice were fasted overnight then IP injected with saline or 125µmol/kg propargylglycine (PPG), a γ-cystathionase (γCSE) non-competitive inhibitor, then 4 hours later in the early light phase IP injected with saline or 2mmol/kg L-cysteine. In study 3, mice were fasted overnight then IP injected with saline or 20mg/kg o-carboxymethyl hydroxylamine hemihydrochloride (CHH), a general inhibitor of pyridoxal 5’ phosphate (PLP)-dependent enzymes, which include cystathionine β-synthetase (CBS), γCSE, cysteine amino transferase (CAT), and cysteine lyase (CL), then 4 hours later IP injected with saline or 2mmol/kg L-cysteine. All enzyme inhibitors have previously been shown to inhibit activity within their respective timeframes and did not affect food intake when given alone.

**Intracerebroventricular (ICV) cannulation.** Rats were implanted with a permanent 22-gauge stainless steel cannula, by a method previously described , projecting into the lateral ventricle. Co-ordinates used for the cannula placement were 0.5mm posterior, +1.5mm lateral and 3.5mm ventral. Co-ordinates were taken from the Paxinos and Watson atlas. Correct cannula placement was verified with a positive dipsogenic response to 150ng Angiotensin II.

Test substances were administered in a volume of 5µl at a rate of 120µl/hour to conscious, free moving animals via a 26-gauge stainless steel injector projecting 1mm beyond the tip of the cannula. Following injection, animals were returned to their individual cage with a pre-weighed amount of food and free access to water.

**Effect of ICV L-cysteine on food intake.** Overnight fasted rats received a single ICV injection of 0.9% Saline (control), L-Cysteine (1 or 2µmol) or 3nmol NDP-MSH (positive control) during the early light phase between 09.00 and 10.00hr (n=5-8). Food intake was measured 1, 2, 4, 8 and 24 hours after administration.

**NMDA receptor and L-cysteine.** Rats were fasted overnight and then received a single ICV injection of saline, 200nmol D-AP5 (a competitive NMDA receptor antagonist, Tocris), 2µmol L-cysteine or 200nmol D-AP5 plus 2µmol L-cysteine (n=5-9). Rats were then returned to their home cages with a pre-weighed amount of chow and food was reweighed at 1, 2, 4, 8 and 24 hours post-administration.

**Exclusion criteria for clinical studies.** All subjects participated in a screening visit, which included a medical history, physical examination, and blood tests to exclude existing medical conditions. Individuals with a history of smoking or drug abuse, heavy alcohol consumption, medical illness (current or previous), inborn errors of metabolism, current pregnancy or breastfeeding, history of allergies, those who were regular blood donors as well as those taking medications known to influence metabolism were excluded.

**Supplementary Results**

**L-cysteine does not mediate its anorectic effects via the NMDA receptor, GPRC6a or via downstream metabolites** Inhibiting the production of glutathione from L-cysteine with the γGCS enzyme inhibitor BSO (19) did not attenuate the effect of intraperitoneal administration of 2mmol/kg L-cysteine on 0-1hr food intake (Supplementary Fig. 2B).

Inhibiting the enzyme γCSE, which catalyzes the production of pyruvate, ammonia and hydrogen sulphide from L-cysteine, with PPG (20, 21) did not attenuate the effect of intraperitoneal administration of 2mmol/kg L-cysteine on 0-1hr food intake (Supplementary Fig.2C).

CHH, an inhibitor of PLP-dependent enzymes (22), which include CBS, γCSE, CAT, and CL, potentiated the effect of intraperitoneal administration of 2mmol/kg L-cysteine on 0-1hr food intake (p<0.05) (Supplementary Fig. 2D). These data suggest that it is L-cysteine itself, rather than downstream metabolites, which suppresses appetite.

We investigated whether the anorectic effects of L-cysteine administration were mediated by the NMDA receptor using the specific NDMA receptor antagonist MK-801. A dose of 10µg/kg MK-801 has previously been shown to block NMDA-receptor mediated effects on food intake (32). However, this dose did not attenuate the effect of L-cysteine on food intake (Supplementary Fig. 2E).

L-cysteine has high affinity for GPRC6A (33), a promiscuous amino acid sensing receptor expressed in the liver, brain, stomach, small intestine and pancreas amongst other tissues (34-36). We therefore investigated whether GPRC6A had a role in mediating L-cysteine’s effect on food intake. Oral and IP administration of L-cysteine significantly reduced food intake to a similar extent in both wild type mice and mice lacking the GPRC6A, demonstrating that GPRC6A is not necessary for the effect of L-cysteine on food intake (Supplementary Fig. 3A and B).

**Supplementary Figure 1.** The effect of intraperitoneal administration of saline or 2mmol/kg L-cysteine at 09.00 on *(A)* VO_2_, *(B)* VCO_2_, *(C)* RER, *(D)* heat, *(E)* XTOT, *(F)* XAMB and *(G)* ZTOT in overnight fasted male C57BL/6 mice in a 22 cage-oxymax CLAMS system (n=10-12). *p<0.05, **p<0.01, ***p<0.001

**Supplementary Figure 2**. The role of downstream metabolites and the NMDA receptor. *(A)* Pathways of L-cysteine degradation and enzymes involved. The effect of *(B)* pre-treatment with 1mmol/kg BSO (γ-GCS inhibitor) (n=9-10), *(C)* pre-treatment with 125µmol/kg PPG (γ-CSE inhibitor) (n=10), *(D)* pre-treatment with 20mg/kg CHH (PLP-dependent enzyme inhibitor) (n=10), *(E)* pre-treatment with 10 or 100µg/kg MK-801 (an NMDA receptor antagonist) on L-cysteine (2mmol/kg IP) induced anorexia in the 0-1 hour period after administration in overnight fasted male mice (n=5-8). All data expressed as mean + SEM. *p<0.05, **p<0.01, ***p<0.001

**Supplementary Figure 3**. GPRC6a is not necessary for the effect of L-cysteine of food intake. *(A)* The effect of oral gavage administration of water and 8mmol/kg L-cysteine on 0-1 hour food intake in wild type and GPRC6a knockout mice (n=5). *(B)* The effect of intraperitoneal administration of saline and 4mmol/kg L-cysteine on 0-1 hour food intake in wild type and GPRC6a knockout mice (n=6). *p<0.05, **p<0.01, ***p<0.001

**Supplementary Figure 4**. NMDA receptor mediates the effect of central administration of L-cysteine on food intake in rats. *(A)* The effect of ICV administration of L-cysteine on 0-1hour food intake in overnight fasted male Wistar rats. *(B)* Effect of ICV administration of 200nmol of the NMDA antagonist D-AP5 on 0-1 hour food intake following central administration of 2 µmol L-cysteine . *p<0.05, **p<0.01

**Supplementary Figure 5.** L-cysteine does not stimulate the secretion of the anorexigenic gut hormones GLP-1 and PYY. The change in plasma (A) GLP-1 and (B) PYY following oral ingestion of vehicle, 0.07g/kg L-cysteine or 0.07g/kg glycine (n=7). Data expressed as mean + SEM

**Supplementary Figure 6.** L-cysteine does not result in anxiety, irritability, nausea, sleepiness, or feelings of warmth in humans. A-E) Visual Analogue scales and area under the curve following ingestion of vehicle, 0.07g/kg L-cysteine or 0.07g/kg glycine (n=7). Data expressed as mean + SEM.

**Supplementary Table 1**. Observed behaviour following oral administration of L-cysteine in rats. Number of observed behaviours expressed as median and interquartile range in male Wistar rats in the 0-1 hr period following oral gavage of water or 4mmol/kg L-cysteine. n=4-10, *p<0.05

**Supplementary Table 2**. Observed behaviour following intraperitoneal administration of L-cysteine in rats. Number of observed behaviours expressed as median and interquartile range in male Wistar rats in the 0-1 hr period following intraperitoneal administration of saline or 2mmol/kg L-cysteine. n=9-10

**Supplementary Table 3**. Observed behaviour following intraperitoneal administration of L-cysteine in mice. Number of observed behaviours expressed as median and interquartile range in male C57BL/6 mice in the 0-1 hr period following intraperitoneal administration of saline or 2mmol/kg L-cysteine. n=7-8, **p<0.01

1. Westerterp-Plantenga MS, Lejeune MP, Nijs I, van Ooijen M, Kovacs EM. High protein intake sustains weight maintenance after body weight loss in humans. Int J Obes Relat Metab Disord. 2004;28(1):57-64.

2. Semjonous NM, Smith KL, Parkinson JR, Gunner DJ, Liu YL, Murphy KG, et al. Coordinated changes in energy intake and expenditure following hypothalamic administration of neuropeptides involved in energy balance. Int J Obes (Lond). 2009;33(7):775-85.

3. Parsons RB, Waring RH, Ramsden DB, Williams AC. In vitro effect of the cysteine metabolites homocysteic acid, homocysteine and cysteic acid upon human neuronal cell lines. Neurotoxicology. 1998;19(4-5):599-603.

4. Reddy DS, Kulkarni SK. Role of GABA-A and mitochondrial diazepam binding inhibitor receptors in the anti-stress activity of neurosteroids in mice. Psychopharmacology (Berl). 1996;128(3):280-92.

5. Ma QP, Allchorne AJ, Woolf CJ. Morphine, the NMDA receptor antagonist MK801 and the tachykinin NK1 receptor antagonist RP67580 attenuate the development of inflammation-induced progressive tactile hypersensitivity. Pain. 1998;77(1):49-57.

6. Standeven AM, Wetterhahn KE. Tissue-specific changes in glutathione and cysteine after buthionine sulfoximine treatment of rats and the potential for artifacts in thiol levels resulting from tissue preparation. Toxicol Appl Pharmacol. 1991;107(2):269-84.

7. Uren JR, Ragin R, Chaykovsky M. Modulation of cysteine metabolism in mice--effects of propargylglycine and L-cyst(e)ine-degrading enzymes. Biochem Pharmacol. 1978;27(24):2807-14.

8. Kim SK, Kim YC. Effect of propargylglycine on synthesis of glutathione in mice. Nutrition Research. 2001;21(10):1373-81.

9. Wallace JL, Vong L, McKnight W, Dicay M, Martin GR. Endogenous and exogenous hydrogen sulfide promotes resolution of colitis in rats. Gastroenterology. 2009;137(2):569-78, 78 e1.

10. Geng MY, Saito H, Katsuki H. Effects of vitamin B6 and its related compounds on survival of cultured brain neurons. Neurosci Res. 1995;24(1):61-5.

11. Seal LJ, Small CJ, Dhillo WS, Stanley SA, Abbott CR, Ghatei MA, et al. PRL-releasing peptide inhibits food intake in male rats via the dorsomedial hypothalamic nucleus and not the paraventricular hypothalamic nucleus. Endocrinology. 2001;142(10):4236-43.

Supplementary Figures

Supplementary Figure 1


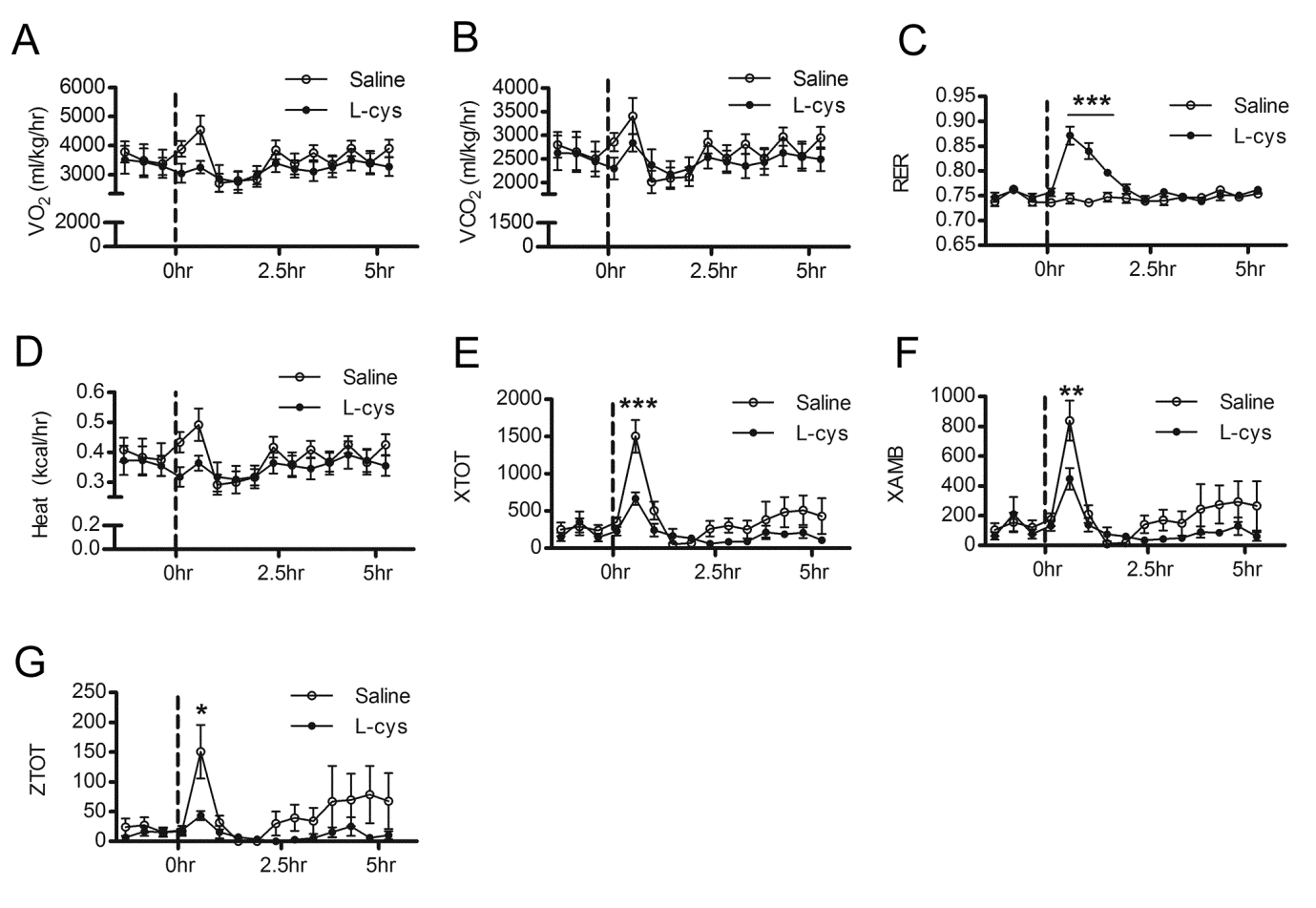


Supplementary Figure 2


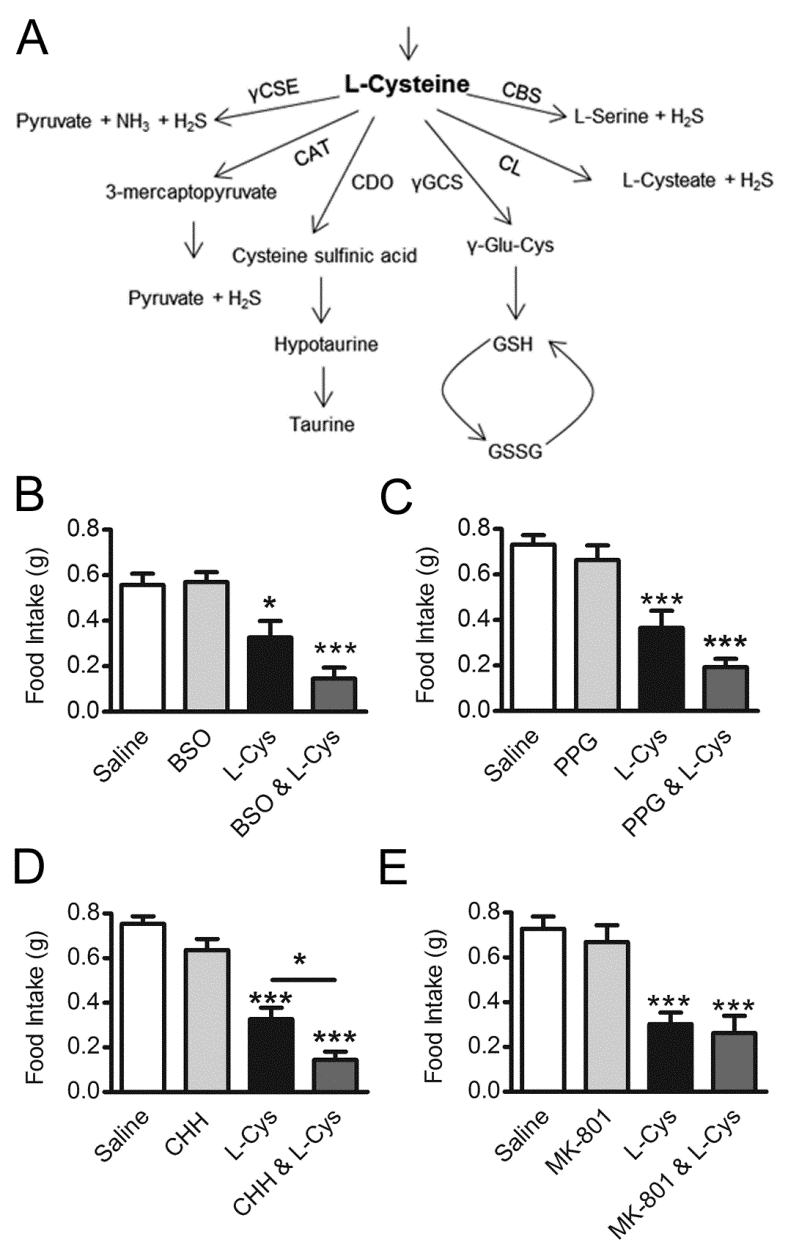


Supplementary Figure 3


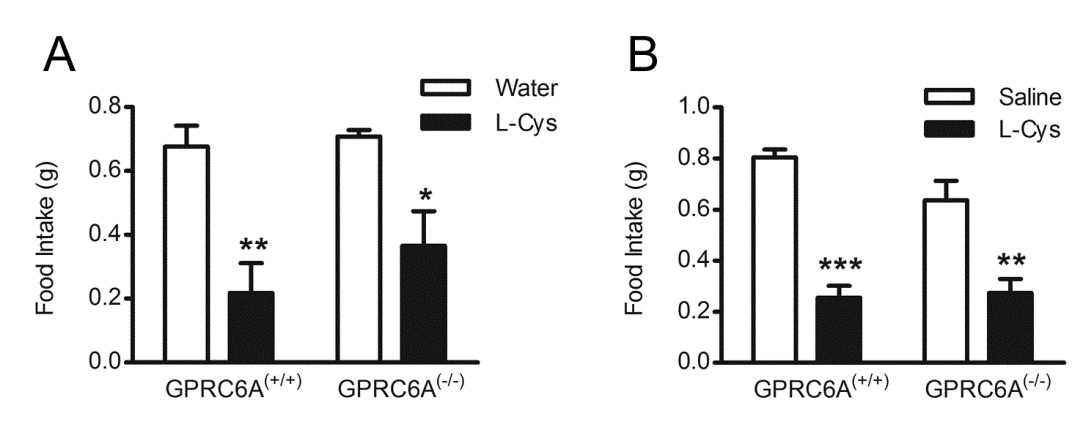


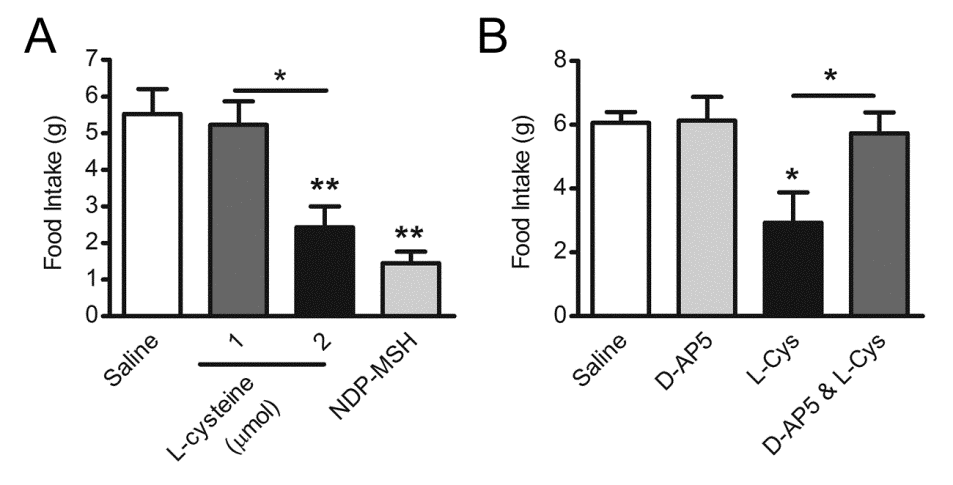
Supplementary Figure4

Supplementary Figure 5


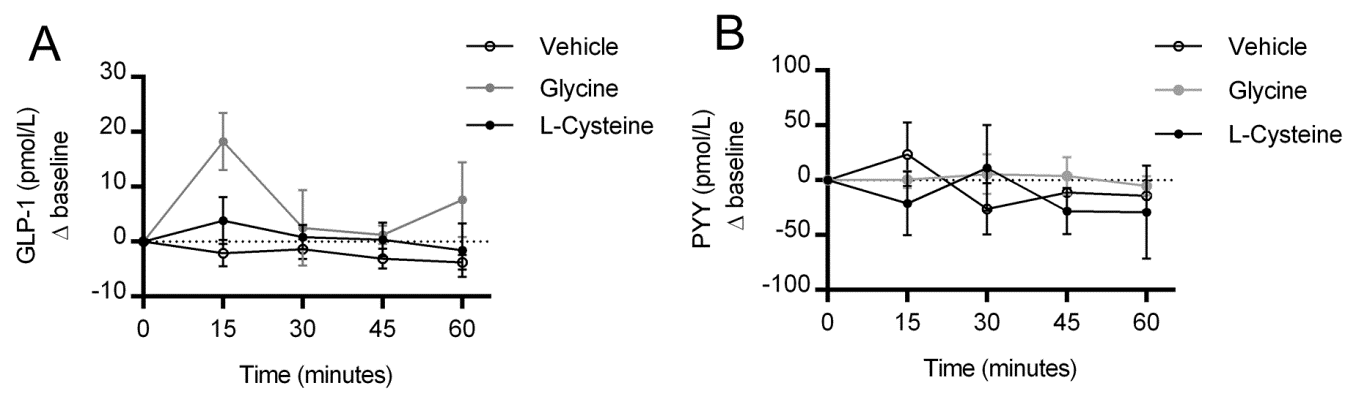


Supplementary Figure 6


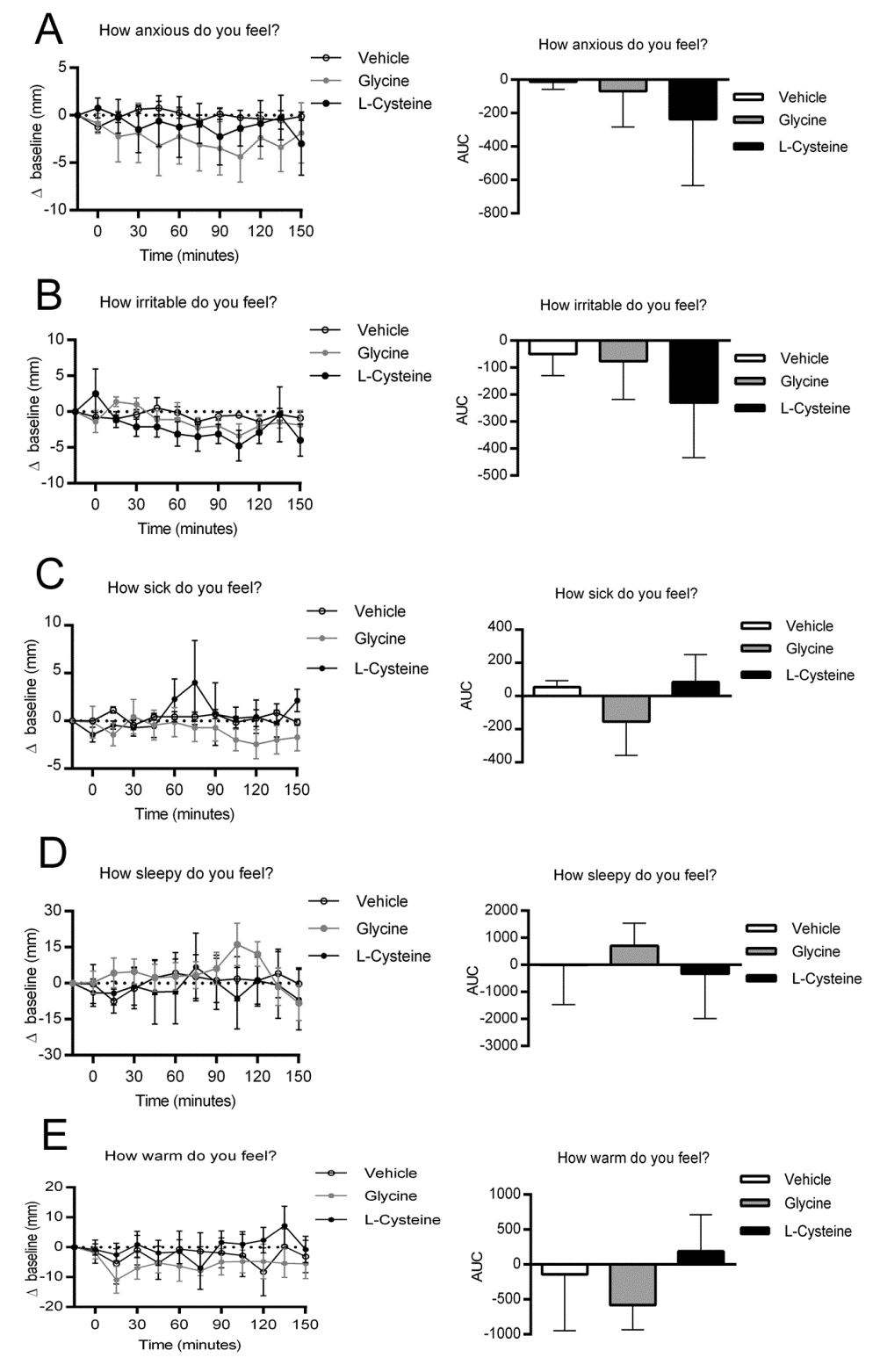


**Supplementary Table 1**

|  | Feeding | Locomotion | Grooming | Resting | Head Down | Pica |
| --- | --- | --- | --- | --- | --- | --- |
| Water | 23.0 (18.75-24.0) | 9.0 (6.75-10.0) | 2.0 (0.0-3.25) | 2.0 (0.0-5.5) | 0.0 (0.0-0.0) | 0.0 (0.0-0.0) |
| L-Cys | 13.0 (9.75-17.0)* | 11.5 (7.0-17.5) | 3.0 (0.5-4.75) | 2.5 (0.25-12.25) | 1.5 (0.0-5.25) | 0.0 (0.0-2.25) |

Supplementary Table 2

|  | Feeding | Locomotion | Grooming | Resting | Head Down | Pica |
| --- | --- | --- | --- | --- | --- | --- |
| Saline | 21.0 (11.5-21.0) | 11.0 (5.0-18.5) | 0.0 (0-3.25) | 3.0 (0.0-6.0) | 0.0 (0.0-3.0) | 0.0 (0.0-0.0) |
| L-Cys | 17.0 (9.5-22.0) | 10.0 (7.0-13.5) | 0.0 (0.0-2.0) | 3.0 (1.5-7.0) | 3.0 (0.0-4.5) | 0.0 (0.0-3.0) |

Supplementary Table 3

|  | Feeding | Locomotion | Grooming | Resting | Head Down | Pica |
| --- | --- | --- | --- | --- | --- | --- |
| Saline | 14.0 (11.25-19.25) | 11.0 (7.0-13.25) | 2.0 (1.75-8.25) | 3.0 (2.75-9.25) | 0.0 (0.0-0.0) | 0.0 (0.0-2.0) |
| L-Cys | 4.0 (1.0-8.0)** | 11.0 (5.0-13.5) | 4.0 (2.0-8.5) | 15.0 (10.5-20.0)** | 0.0 (0.0-0.0) | 0.0 (0.0-0.0) |
